# Supplementary figures and images for: Essential role of the conserved oligomeric Golgi complex in Toxoplasma gondii
Source: mBio. 2023 Nov 15;14(6):e02513-23. doi: 10.1128/mbio.02513-23 (PMC10746232; doi:10.1128/mbio.02513-23)

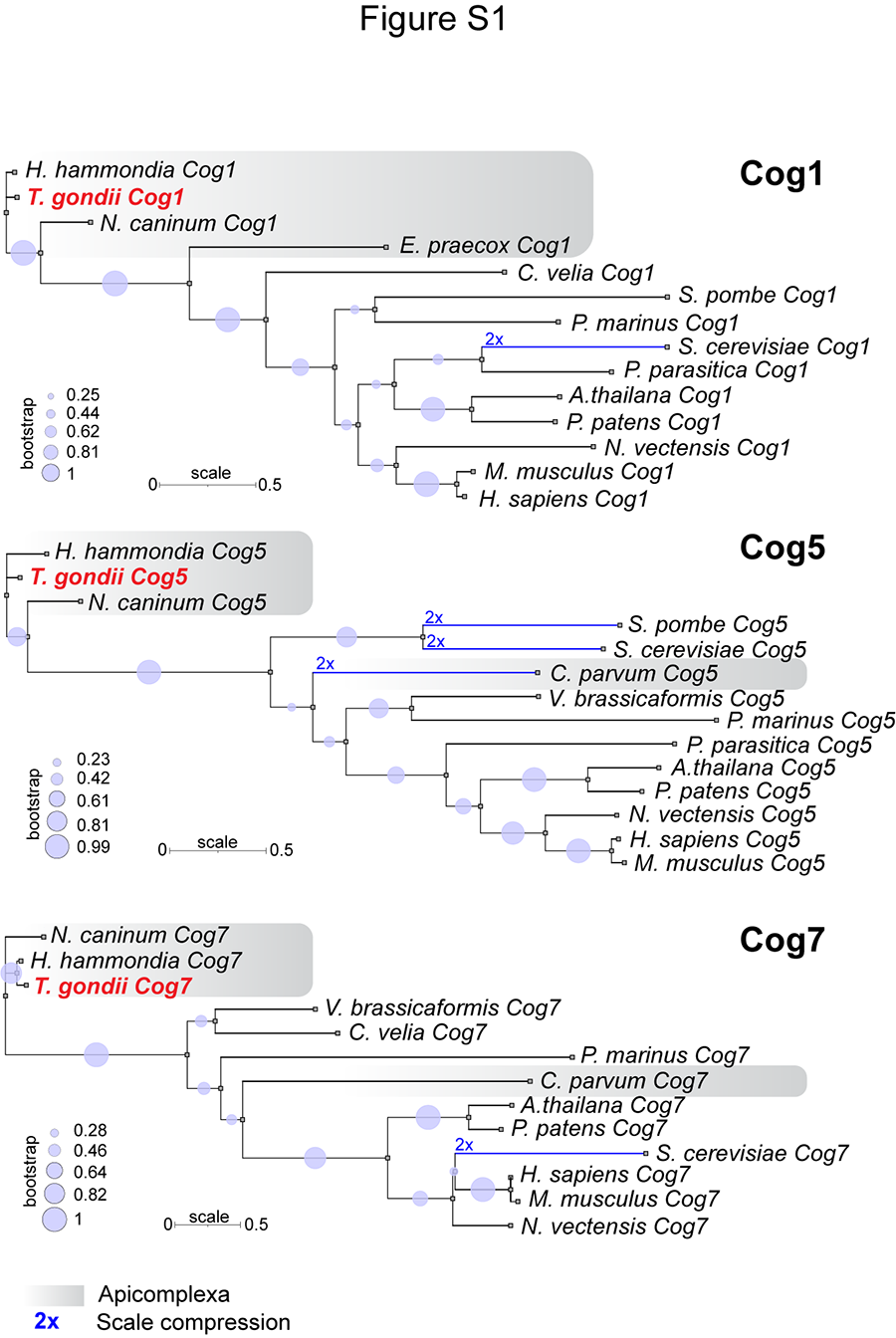

Supplement: Figure S1 — Phylogenetic analysis of Toxoplasma COG complex subunits. [file mbio.02513-23-s0001.tif]

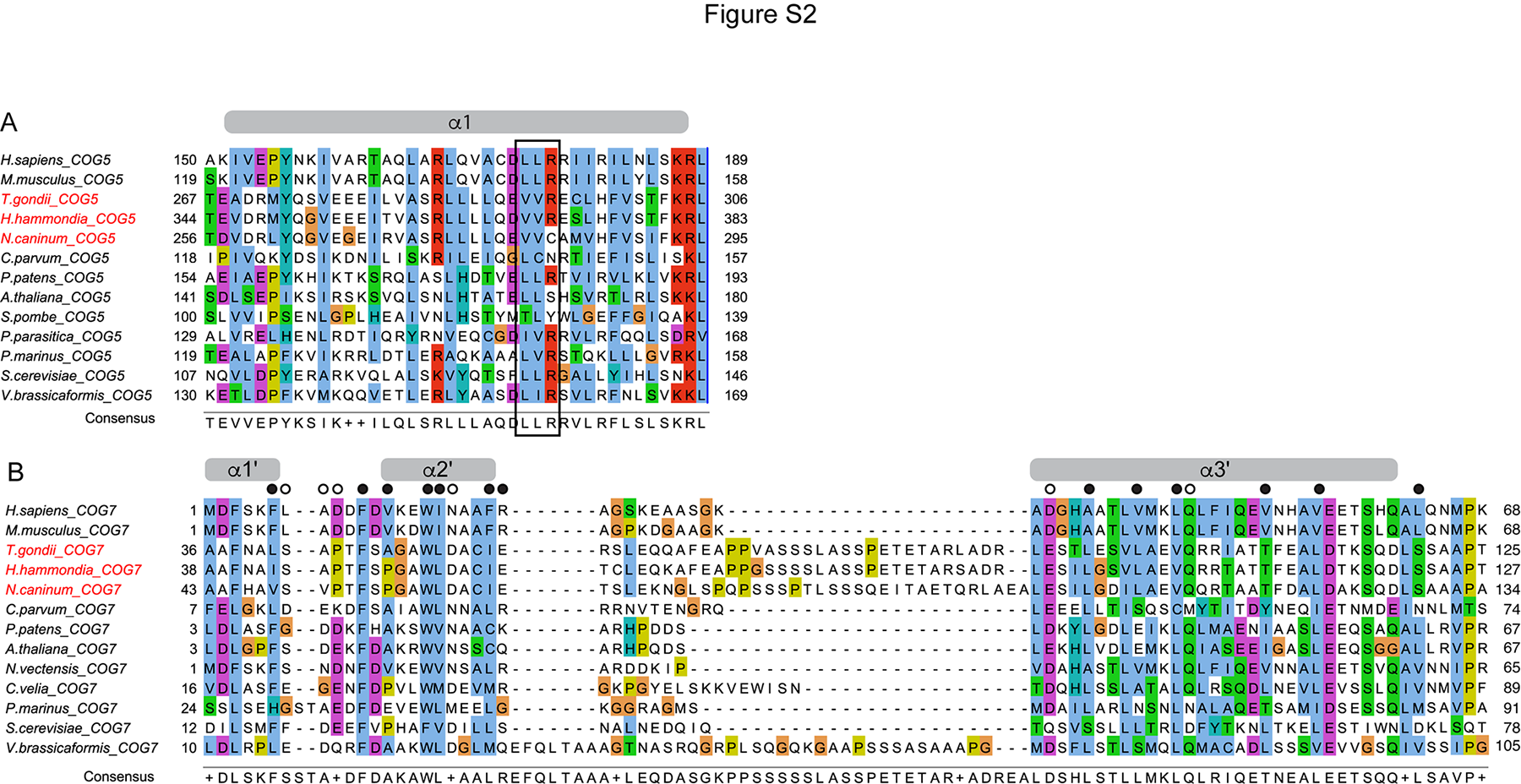

Supplement: Figure S2 — TgCog5 and TgCog7 signature domains. [file mbio.02513-23-s0002.tif]

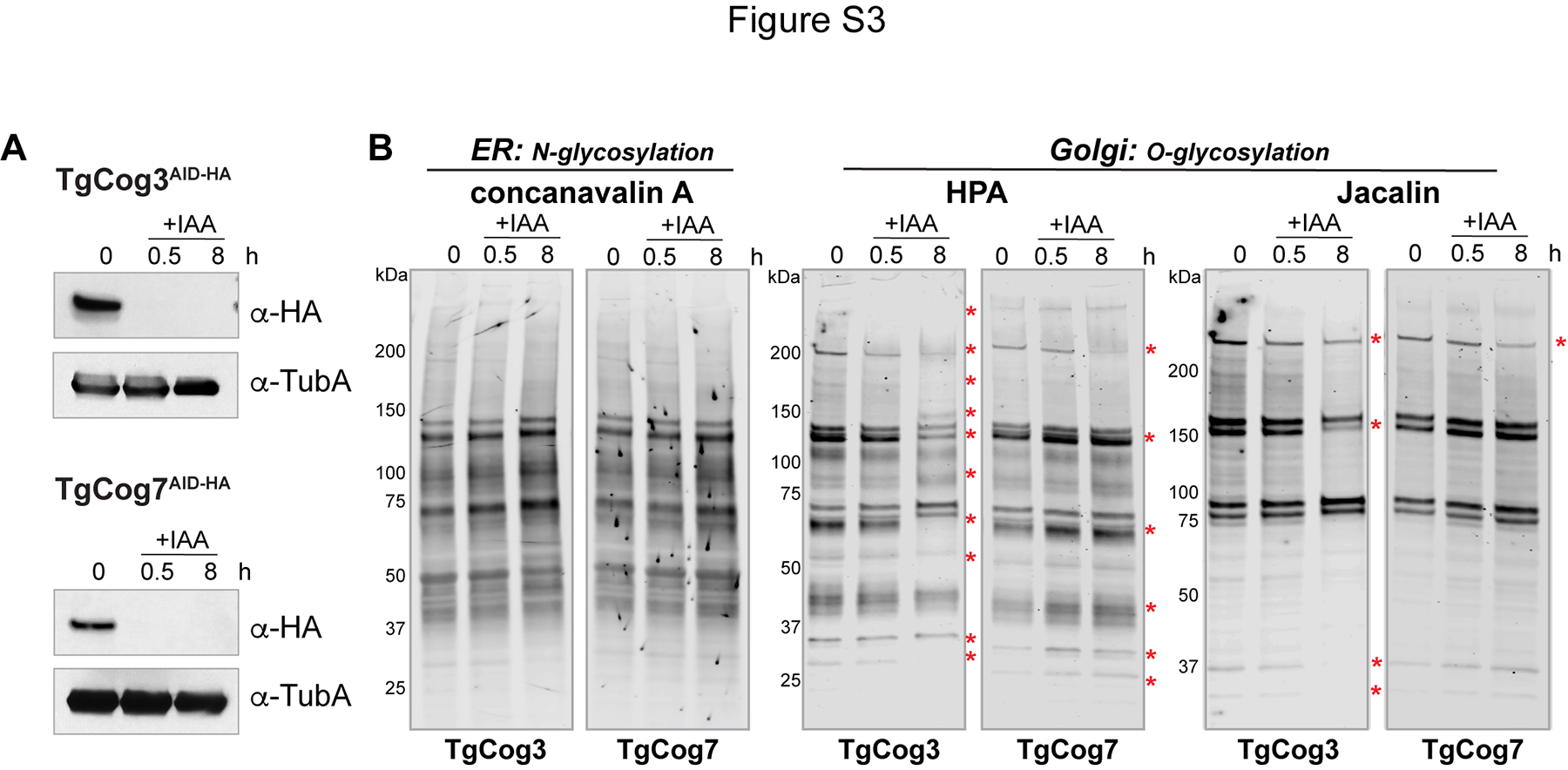

Supplement: Figure S3 — Toxoplasma Cog complex deficiency affects protein glycosylation in the Golgi. [file mbio.02513-23-s0003.tif]

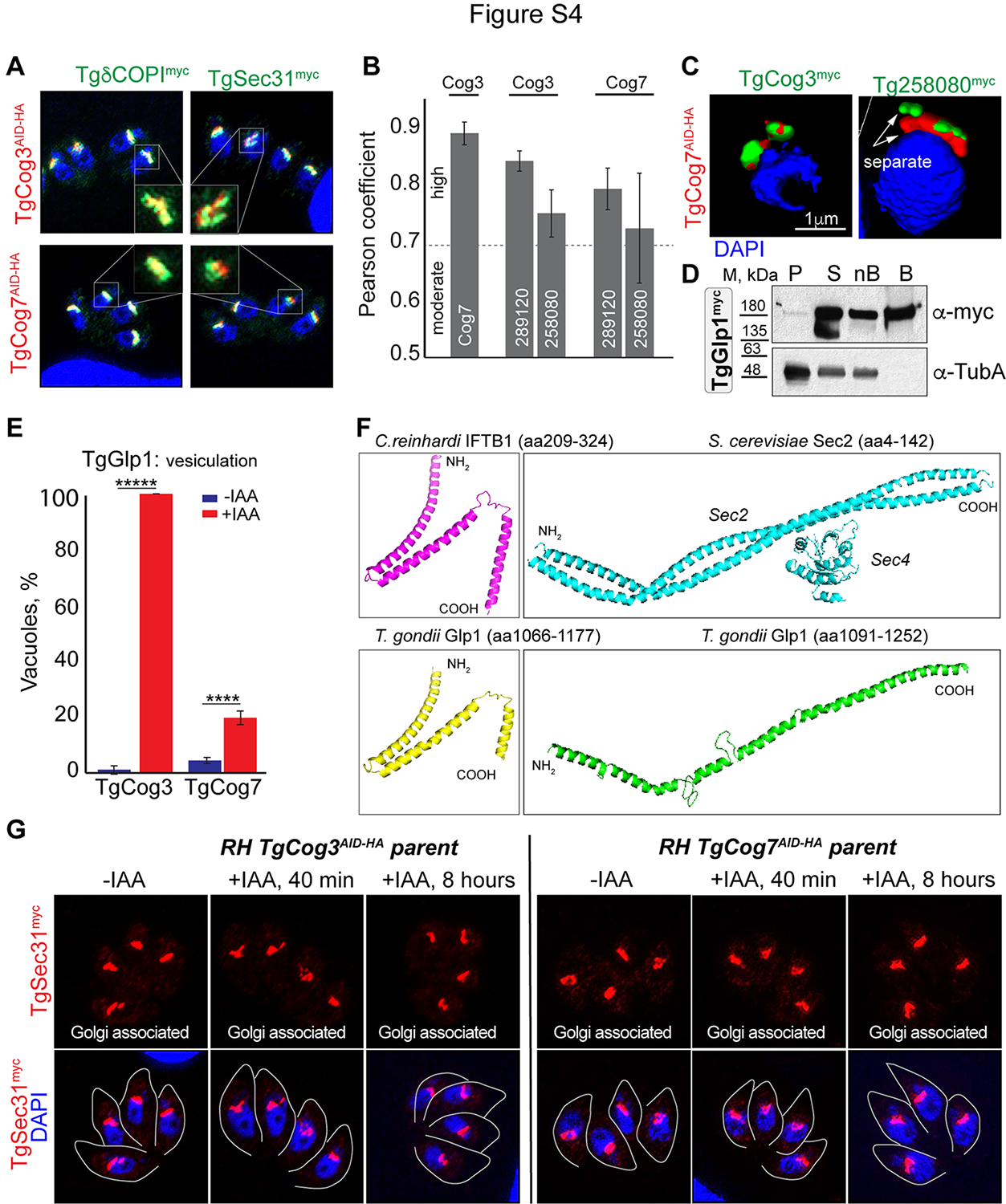

Supplement: Figure S4 — Analysis of the T. gondii COG complex interactions. [file mbio.02513-23-s0004.tif]
