# Supplementary material for: Essential role of the conserved oligomeric Golgi complex in Toxoplasma gondii
Source: mBio. 2023 Nov 15;14(6):e02513-23. doi: 10.1128/mbio.02513-23 (PMC10746232; doi:10.1128/mbio.02513-23)
Supplement: Supplemental legends — Legends to Fig. S1 to S4 and Tables S1 to S4. [file mbio.02513-23-s0005.docx]

SUPPLEMENTAL FIGURE LEGENDS

**Figure S1. Phylogenetic analysis of Toxoplasma COG complex subunits TgCog1, TgCog5 and TgCog7.** Shaded blocks show apicomplexan orthologs. Blue line is a compressed tree branch, the compression coefficient is indicated.

**Figure S2. The TgCog5 and TgCog7 signature domains.**

**A.** Alignment of the COG5 CATCHR domain (MUSCLE). Secondary structure (above) and sequence conservation (below) are shown, including central LLR (VVR) motif. **B.** Alignment of the COG7 α1’, α2’, and α3’ helixes that are involved in interaction with COG5 CATCHR domain (MUSCLE). Critical amino acid residues are marked with closed (non-polar interaction) and open (polar interaction) circles.

**Figure S3. The *Toxoplasma* Cog complex deficiency affects protein glycosylation in Golgi.**

**A.** Western Blot analysis confirmed efficient downregulation of TgCog3^AID-HA^ and TgCog7^AID-HA^ after 30 min and 8 hours of IAA treatment. Western blots were probed with α-HA to detect the COG complex subunits, and with α-Tubulin A to verify equal loading of the total lysates.

**B.** Images of the lectin-binding analysis. Total lysates of the IAA treated and not treated tachyzoites were probed with Concanavalin A, HPA or Jacalin. Detected major changes are indicated with red stars.

**Figure S4. Analysis of the *T. gondii* COG complex interactions.**

**A.** immunofluorescent microscopy analysis of the parasites expressing TgδCOPl^myc^ or TgSec31myc in the RH TgCog3^AID-HA^ or TgCog7^AID-HA^ mutants. Parasites were co-stained with α-myc (green), α-HA (red) and DAPI (blue). Insets shows the proteins overlap in the Golgi region. **B.** Pearson coefficients of TGME49_258080, TGME49_289120 proteins colocalization with the COG complex subunits TgCog3 and TgCog7. The mean and the SD values collected from a minimum 10 parasites are plotted on the bar graph. **C.** A 3-D reconstruction of the immunofluorescent microscopy images of the parasites expressing TgCog3^myc^ or Tg258080^myc^ in the RH TgCog7^AID-HA^ line. Parasites were co-stained with α-myc (green), α-HA (red) and DAPI (blue). **D.** Western Blot analysis of immunoprecipitated TgGlp1^myc^ complexes. The insoluble [P, pellet], soluble [S], depleted soluble fractions [nB, not bound] and the beads with precipitated complexes [B] (10 times more than the other fractions) were probed with α-myc and α-Tubulin A antibodies. **E.** Quantification of the parasites that showed Golgi vesiculation (TgGlp1^myc^ marker) upon 8-hour treatment with IAA. The mean of the vacuole counts, and the SD values are plotted on the graph. The raw and the t-test values are in Table S3. **F.** Folding prediction for selected regions of *T. gondii* Glp1 (bottom panel) and models (top panel) are shown (PyMol). Note that S. *cerevisiae* Sec2 shown in the complex with Sec4. **G.** immunofluorescent microscopy analysis of TgSec31myc in parasites expressing (-IAA) or lacking TgCog3^AID-HA^ or TgCog7^AID-HA^ (+IAA). TgSec31 was detected with α-myc antibodies and co-stained with DAPI (blue) and α-TglMC1 (traced with grey line).

**Table S1.** Primers and transgenic strains used in the study.

**Table S2.** The Cog5 and Cog7 orthologs (phylogeny).

**Table S3.** Raw counts for Figures 5 and S4.

**Table S4.** SAINT analysis of TgCog8, TgUlp1 and TgGlp1 proteomes.
